# Technology-Enabled Workplace Learning Through Rethinking Electronic Health Records to Support Performance Feedback: Protocol for a Mixed Methods Study

**DOI:** 10.2196/66824

**Published:** 2025-05-23

**Authors:** Anna Janssen, Mia Nazir

**Affiliations:** 1 School of Medical Sciences Faculty of Medicine and Health The University of Sydney Sydney, New South Wales Australia

**Keywords:** digital health, electronic health records, reflective practice, professional learning, technology, digital technology, EHR, mixed methods

## Abstract

**Background:**

The health sector collects a plethora of electronic health data via digital technologies, such as electronic health records (EHRs) and electronic medical records (EMRs). The primary use of EHRs includes supporting service delivery, providing data on patient information, and health care operations. Secondary uses of these systems can include quality improvement activities and research, and possibly inform policy. One underexplored secondary use of data from these systems is to enable health care professionals to understand their performance, reflect on their practice, and potentially support enhanced professional and workplace learning. There is growing interest and an increase in policies to focus on motivating the use of this type of data as part of mandatory Continuing Professional Development. Despite this, the design of EHRs is not conducive to the use of these systems for reflective practice, and there are few best practice guides for how to scaffold the use of these data for secondary use.

**Objective:**

The aim of this project is to determine how EHRs and EMRs can be leveraged to enable formative performance feedback for health care professionals. The primary objective is to explore the use of these systems by health care professionals to further understand the current and possible future use of these records for reflective practice, performance feedback, and workplace learning.

**Methods:**

The project will use a mixed methods design to enable a holistic picture of participant behaviors. Study data are being collected over 3 phases. Phase 1 consists of interviewing health care professionals and clinicians about their experiences with EHRs and EMRs. Phase 2 will involve surveying health care professionals about specific EHR features, and phase 3 will encompass workshopping discussions around EMR functionality and design with key informants. Participants for phases 1 and 2 will be a convenience sample of health care professionals who self-select and volunteer to participate in the study. Participants for phase 3 will consist of policy makers, representatives from peak bodies, technology vendors, health care professionals, and others. Data from phase 1 will be thematically analyzed to identify key features of EMR and EHR design for prioritization in phase 2. Phase 2 survey responses will be descriptively analyzed to understand the most important features in record design to support reflective practice. Phase 3 workshop data will be thematically analyzed to identify design insights for EHRs and EMRs that support professional learning.

**Results:**

The project is currently in its interview phase and is expected to publish results in mid-2025.

**Conclusions:**

The project will generate new knowledge on the extent to which data collected by workplace technologies provide health care professionals with formative performance feedback. It will also develop a conceptual design for EHRs that supports health care professional learning, which could be leveraged by developers of these technologies in future implementations.

**International Registered Report Identifier (IRRID):**

DERR1-10.2196/66824

## Introduction

### Background

The modern health sector is highly digitized [1]. As a result, health care professionals, consumers, and other stakeholders interact with a plethora of commonly used digital health technologies [2-4]. One widely used technology in the health sector is electronic health records (EHRs) and electronic medical records (EMRs), which are types of clinical information systems that serve as sources of patient health information. They are designed for use by health care professionals to capture data reflecting specific clinical experiences [5]. The data that clinical information systems including EHRs and EMRs collect have great potential for a range of secondary applications, including research, informing policy, and quality improvement [6,7]. However, these systems are currently underused for secondary applications such as Continuing Professional Development (CPD), practice reflection, personalized workplace learning, and insights about their performance [8,9].

CPD, reflective practice, and lifelong learning are essential activities for health care professionals to stay up to date on the latest evidence and maintain their registration to practice [1]. Health care professionals engage in a variety of learning activities, including attending local or international conferences, participating in mentorship and peer review, joining seminars, journal clubs, and other activities [10]. A large portion of these activities also takes the form of workplace learning [11]. The workplace provides a unique environment and a unique instructional context that is often focused on informal and experiential learning [12] to help overcome workplace problems or improve performance [12]. It can also offer a range of formal and informal pathways for receiving performance feedback. Although much of the learning that health care professionals engage in takes place in the workplace, there is a dearth of research on how to align learning activities with the clinical environment and on the pedagogical value of doing so. One mechanism to support data-driven professional development and enable better-aligned workplace learning is the utilization of data from EHRs and EMRs for the personalization of digital workplace education, data-driven CPD, or supporting reflective practice [8].

EHRs are sources of electronic health data that are increasingly being implemented in health care [13]. Existing enterprise-level systems focus on the use of their data for immediate patient care and billing, with a particular focus on recording patient information, including patient demographics, progress notes, medication, and laboratory results, which is generated by one or more encounters in any health care delivery setting [5]. The primary intent of EHRs is to provide data on patient care and health care operations; however, there is an increasing desire to explore the secondary use of data from clinical information systems in nondirect care operations, such as research, analytics, and measurements of quality and safety. In this context, they have been used for projects, such as research using population-wide data on cancer patient experiences and therapy outcomes [6], and quality improvement activities, such as toxicity monitoring and symptom management of chemotherapy treatments [7].

One underexplored secondary use of EHRs and EMRs is to support health care professional learning, performance review, and reflective practice. Reflective practice is the process by which professionals review and reflect on past experiences to gain understanding and identify opportunities for potential improvement [14]. While modern EHRs increasingly incorporate feedback through clinical decision support systems and dashboards, less work has focused on how EHRs can more effectively use onboard data to support reflective practice and health care professional learning. However, interest in better using clinical information systems is increasing, with a small but growing body of research in an area termed “Practice Analytics” [15-17]. Practice analytics seeks to equip health care professionals, their workplaces, and their associated peak bodies with the data and tools needed to review their own clinical practice [16]. Literature related to the use of electronic health data to date indicates that, when used correctly, it can help health care professionals understand their performance [7]. Further, it indicates that health care professionals themselves would like to access these data for learning purposes [9]. However, it has also been noted that health care professionals currently have varied access to these data for understanding their performance, and there is considerable variation in the ways it is visualized and shared with them [18].

### Study Objective

The aim of this project is to determine how EHRs and EMRs can be leveraged to enable formative performance feedback for health care professionals. Over the course of the project, the researchers aim to understand how health care professionals are currently using EMRs and EHRs to support their practice, what the role of these technologies is in performance feedback and reflective practice of medical practitioners, and how the design of these technologies can be rethought to support a “next-generation” EHR that could support reflective practice. This project has been designed to build upon existing literature in the field of practice analytics and beyond, indicating that clinical information systems could better facilitate health care professional learning [19]; however, current solutions are not well-designed to do so [20]. This work is particularly timely, as global work is being undertaken to develop next-generation EHRs and EMRs [21]. This provides a unique opportunity to design new systems that incorporate interface design and scaffolds, ensuring that EHRs not only use data for direct patient care but also provide data back to health care professionals and teams for performance review and to support evidence-based practice. There is not only technological innovation driving change in this space but also an evolution of the policy landscape regarding how health care professionals engage in learning activities. Policies are increasingly encouraging health care professionals to better use electronic data collected from repositories, such as EHRs, as a component of mandated CPD and for revalidation [22].

## Methods

### Study Design

A mixed methodology is being used to undertake the project, as this methodology is the most appropriate for answering the individual aims of the study. Mixed methodologies are particularly beneficial for balancing out the limitations of qualitative and quantitative methodologies and are particularly important for getting a holistic picture of participant behaviors [23]. The methodology is particularly aligned with the project described in this proposal, as it will not only enable the collection of data on what people do but also provide a rich understanding of why these behaviors occur [23].

**Figure 1:**
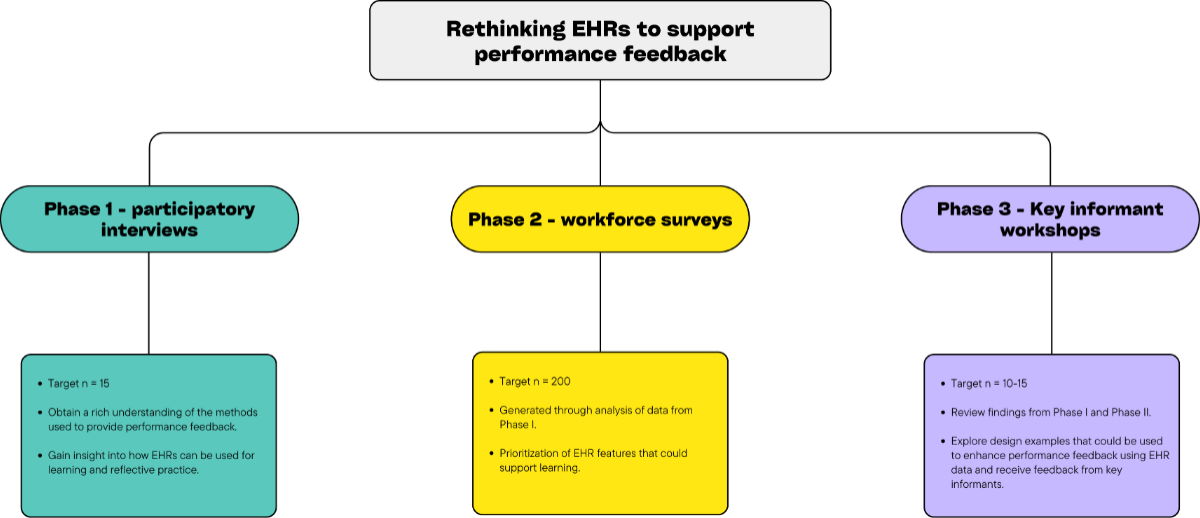
Study design. EHR: electronic health record.

The project will be undertaken in 3 phases: needs finding (phase 1), prioritization (phase 2), and key informant consultation (phase 3). For an overview of the research flow, refer to Figure 1*.* The protocol follows recommendations listed in the TIDieR (Template for Intervention Description and Replication) checklist [24] (Multimedia Appendix 1).

### Participant Recruitment and Sample Size

#### Phases 1 and 2

Potential participants for phases 1 and 2 will be a convenience sample consisting primarily of health care professionals from Australia who self-select and volunteer to participate in the study. Although, health care professionals from other countries who use EMRs and speak English are also eligible to be interviewed. All professions listed as eligible are accredited by the Australian Health Practitioner Regulation Agency [25] and are eligible to participate in interviews. To be eligible to participate in the study, health care professionals do not need to use a specific EMR. However, it is anticipated that many will use Oracle [26] or Epic Systems [27], as these are widely used enterprise solutions. For phase 3, potential participants will consist of a purposeful sample of key informants from Australia who volunteer to participate in workshops discussing EMR functionality and design. Key informants will include policy makers, representatives from peak bodies, technology vendors, health care professionals, and others.

A convenience sample method will be used to recruit participants for phases 1 and 2 of the research. Potential participants will be primarily recruited via email notices sharing information about the study and a link to the digital participant information and consent form, circulated by organizations with links to health care professionals.

The researchers will also distribute a link to the survey through their professional social media profiles (examples include Twitter [subsequently rebranded X] and LinkedIn) and professional networks, which include contacts at numerous health care organizations, and ask them if they can share the link with individuals they may know. Participants who consent to participate in phase 1 will have the option to indicate if they wish to be recruited for subsequent phases of the project. All phase 1 participants will be invited to complete the phase 2 survey if they indicate they are happy to be contacted for this purpose; however, it is anticipated that most survey respondents will be a new cohort of health care professionals.

In addition to the methods listed above, snowballing will be used to recruit potential participants for this study. Interviewees who consent to participate in the study will be asked whether they have any suggestions for other individuals we could speak to about the project. Passive snowballing will be used, where interviewees who suggest others for the interview will be asked to forward information about the study to the relevant person, including instructions on how interested individuals can reach out to the research team to participate.

The sample size for phase 1 will be determined by the point at which saturation is reached during the interviews—that is, when no new insights are emerging from interviews. The size at which saturation occurs in qualitative research is debated [28]; however, in this study, we anticipate that 15 participants will be required to achieve saturation of themes for phase 1. At the conclusion of this phase, the researchers aim to have developed a thorough understanding of the methods used to provide performance feedback and to have gained insights into how EHRs can support these approaches.

The target sample size for the survey is 200 complete responses. This sample size has been chosen as it is feasible for recruitment and sufficient to provide enough data for descriptive statistical analysis to identify key insights related to the research questions. At the conclusion of this phase, the researchers aim to have a prioritized list of suggested changes that could be implemented in EHRs to enhance their design to support performance feedback and reflective practice.

#### Phase 3

A purposeful sample will be used to recruit participants for the final phase of the research. Potential participants will be identified through publicly available sources such as organizational websites, as well as through the researcher’s professional networks of individuals who have relevant expertise to participate in the workshops. The names and contact information (email addresses) of key informants who are eligible to participate in the study will be aggregated into an email distribution list.

An initial contact will be made with potential participants on this list by a member of the research team via an email introducing the project and providing information about the co-design workshops. The email will also provide information clarifying how the participants’ email contact information was obtained. Potential participants will be given a detailed participant information sheet to provide them with information about the study and will be given time to consider whether they wish to participate before they opt to complete the internet-based consent form. When completing the internet-based consent form, participants will be given the option to find out more about the study from a member of the research team prior to consenting.

The sample size for phase 3 will be determined by the point at which saturation is reached during the interviews, that is, when no new insights are emerging from the interviews. We anticipate that around 10 participants will be required to achieve saturation for phase 3. At the conclusion of this phase, the researchers aim to have worked examples of how EHRs’ design could be modified or extended to incorporate the features prioritized in the phase 2 survey.

### Data Collection and Procedures

#### Phase 1

In this initial phase of the project, data are being collected via semistructured interviews. Health care professionals who use EMRs as part of their jobs will be recruited via the methods described in the previous section. Each interview is anticipated to take a maximum of 45 minutes. As part of this process, potential participants will be made aware that their involvement in the study is voluntary. The initial email provides potential participants with a link to a digital participant information statement and consent form. The consent form must be signed prior to participation in the study. Consent will be reconfirmed at the beginning of the interview or technology observation session. Participants will have the option to stop the interview at any time and request to withdraw from the study.

Each semistructured interview will be undertaken by a researcher experienced in qualitative methods and informed by an interview guide to ensure consistency of data collection. For the semistructured interview guide, refer to Multimedia Appendix 2*.* Interviews will be conducted using an online videoconferencing tool. The interviews will be audio and video recorded. Audio recordings from the interviews will be transcribed and deidentified by a member of the research team prior to analysis. Each participant will be allocated a code that will be used to deidentify their interview transcript.

#### Phase 2

In the second phase of the project, data will be collected via a secure internet-based survey platform. Health care professionals who use EMRs as part of their jobs will be recruited via the methods described in phase 1. Participants will be provided with a link to access the survey. All responses collected through the survey will be anonymous. It is anticipated that the survey will take approximately 15 minutes to complete. For the prioritization schema that will be used in the survey, refer to Multimedia Appendix 3*.*

#### Phase 3

In the final phase of the project, data are being collected via online and face-to-face workshops. Key informants including health care professionals, technologists, and regulators will be recruited to participate in the workshops using the methods described in the phase 2 workshops are expected to take approximately 60 minutes. For the workshop guide, refer to Multimedia Appendix 4.

Each workshop will be undertaken by researchers experienced in qualitative methods and guided by a consistent workshop outline. The workshops will be audio-recorded. Audio recordings from the workshops will be transcribed and deidentified by a member of the research team prior to analysis. A second researcher will also be present to take detailed field notes of key points of discussion during the workshops.

### Data Analysis

#### Phase 1

Semistructured interviews (phase 1) will be transcribed and deidentified. Braun and Clarke’s [29] thematic analysis techniques will be broadly applied to inform the coding process. Transcripts will be read by the researchers prior to coding to gain a clear understanding of the material. Line-by-line coding of the transcripts will be conducted by each researcher independently and then discussed and reflected on to identify common points and understand subjective variations. Codes will then be grouped by related themes and subthemes with the end goal of creating a summary list of topics that describe key features of EMR and EHR design that support the reflective practice of health care professionals. Iterative discussion between researchers will be used to establish consensus on agreed themes and subthemes and to inform the final list of topics that will be incorporated in the phase 2 survey.

#### Phase 2

Survey responses will be extracted from the internet-based platform, cleaned to remove incomplete responses, and then analyzed in aggregate form. Descriptive analyses will be used to identify key insights related to the research questions from the survey data. This analysis will include identifying which features of EHR and EMR design health care professionals most highly prioritize for integration into a redesigned feature, as well as a potential description of features in current systems that are prohibitive to reflective practice.

As this is a mixed-methods study, integrative analysis of the survey and interview data will also be undertaken to understand key insights from the results [30]. Data from different sources will be triangulated to build a complete picture of how health care professionals use EHRs and EMRs to engage in reflective practice and identify opportunities to enhance the design of these systems to support learning and reflective practice more effectively.

#### Phase 3

Workshop recordings will be transcribed and deidentified. Analysis will be undertaken using methods described for phase 1. Transcripts will be read by the researchers prior to coding to get a good overall sense of the material. Line-by-line coding of the transcripts will be conducted until saturation has been reached. Codes will then be grouped by related themes and subthemes. Iterative discussion between researchers will be used to establish consensus on agreed themes and subthemes.

### Ethical Considerations

The study received ethics approval from the University of Sydney Human Research Ethics Committee (protocol number 2024/HE000253). All participants will provide written informed consent before participating in the study. All data will be deidentified prior to analysis. Participants do not receive compensation for participation in the study.

## Results

As of January 2025, we have interviewed 6 participants. We expect to write up the results by late 2025.

## Discussion

### Expected Findings

This protocol describes a methodology for undertaking a mixed methods study designed to generate insights on how next-generation EMRs and EHRs could better support health care professional learning. The study is undertaking a triphasic approach consisting of phase 1 (needs finding), phase 2 (prioritization), and phase 3 (key informant consultation). This approach is being used to obtain a rich understanding of the complex challenges of designing clinical information systems that support reflective practice, understanding of health care professional performance, and workplace learning. It has been recognized that there is a notable knowledge gap on the secondary use of clinical information systems like EMRs and EHRs for enabling health care professionals to gain insights into their performance [9,20]. Furthermore, policy changes are increasingly encouraging health care professionals to use data from these types of systems to chart data and set goals; however, there are few evidence-based methods for providing feedback to support professional learning and reflective practice [17].

The findings from this study will expand the understanding of the methodologies that can be used effectively in the relatively new field of practice analytics. There is currently only one protocol published presenting a practice analytics methodology [15], which emphasizes the need to use multidisciplinary approaches for delivery on a large-scale program. The study described in this protocol builds on the existing methodological approaches used in practice analytics, incorporating multidisciplinary methods, such as needs finding interviews and key informant workshops, to gain a deeper understanding of how technology design and human interaction with it could be used to enable workplace-based learning in the area of practice analytics. A triphasic approach is being used to identify health care professionals’ needs for EHRs that enable reflective practice and workplace learning, prioritize features that are most conducive to this process, and then aggregate key stakeholder perspectives on how these priorities could be harnessed in a next-generation EHR or EMR design.

Finally, this study aims to generate new insights on how next-generation EHRs and EMRs could be designed to better support performance feedback, workplace learning, and data-driven CPD. The literature to date has identified that health care professionals have an interest in accessing more data about their practice from clinical information systems [9], and there is advocacy for enhancing existing clinical information systems to better facilitate health care professional learning [19]. Despite this interest, there is notable variation in current approaches for visualizing and sharing these data with health care professionals [8], and there are few examples of best-practice approaches for designing clinical information systems that support reflective practice [20]. The study described in this protocol aims to address this gap by generating new knowledge on how one type of clinical information system, EHRs and EMRs, could be better used to support workplace learning, reflective practice, and performance feedback.

### Limitations

A limitation of this study is that we are using a convenience sample, largely recruited from Australia, for the first 2 phases of the study, which may limit the generalizability of our findings across health professions and geographic regions. This limitation is being mitigated by undertaking extensive recruitment via a breadth of channels in an attempt to reach participants with a breadth of insights. An additional potential limitation is not focusing on a single EMR or EHR as part of recruitment for this study, as this may also influence the generalizability of findings. The researchers decided not to take this approach because these records have limited use in supporting reflective practice. We aimed to obtain a broad range of insights into how health care professionals are using these systems, and focusing on only the most commonly used enterprise solutions could exclude valuable insights from bespoke EMRs, EHRs, or smaller systems. Future researchers should consider undertaking a study looking specifically at larger enterprise systems for supporting reflective practice.

### Conclusions

EHRs are increasingly prevalent in the health sector, and their implementation and ongoing support often represent a significant financial investment. They are a unique repository of electronic health data that has a plethora of potential uses in health professions. One underexplored use of EHRs and the data they collect is to enable health care professionals to understand their performance and engage in data-driven learning activities. Increased understanding of the extent to which these workplace technologies provide health care professionals formative performance feedback is important in order to understand opportunities to design and use these technologies more effectively to support reflective practice in the future.

## Appendix

Multimedia Appendix 1 TIDieR (Template for Intervention Description and Replication) checklist.

Multimedia Appendix 2 Interview guide.

Multimedia Appendix 3 Prioritization schema.

Multimedia Appendix 4 Workshop guide.

## Abbreviations

CPD: Continuing Professional Development
EHR: electronic health record
EMR: electronic medical record
TIDieR: Template for Intervention Description and Replication

## Data Availability

The datasets used or analyzed during the study can be made available from the corresponding author upon reasonable request.
