# Supplementary material for: Technology-Enabled Workplace Learning Through Rethinking Electronic Health Records to Support Performance Feedback: Protocol for a Mixed Methods Study
Source: JMIR Res Protoc. 2025 May 23;14:e66824. doi: 10.2196/66824 (PMC12144470; doi:10.2196/66824)
Supplement: Multimedia Appendix 2 [file resprot_v14i1e66824_app2.pdf]

Technology enabled workplace learning: Rethinking Electronic Health Records to support performance feedback

Interview Guide

Demographics

1. What is your health profession?
2. What is your career experience level (early, mid, senior)?
3. Approximately how long have you been working at your current organization?

Use of Electronic Health and Medical Records

4. What Electronic Health Record(s) do you use in your role?
   Prompts:
   - Has your organisation been using this system the entire time you've been working there?

5. Can you please tell me about how you use the Electronic Health Record in your role?

   Prompts:
   - Could you walk me through how you use the Electronic Health Record when you interact with a patient?
   - Could you walk me through the type of information you record using structured fields?
   - Could you walk me through any types of information you recording in unstructured fields?
   - Are there any other points in service delivery where you use the Electronic Health Record?
     - Do you every use the Electronic Health Record as part of multidisciplinary team discussions?
     - Do you ever use the Electronic Health Record to retrospectively review your clinical decisions?

6. What do you find particularly useful about the Electronic Health Record?
   Prompts:
   - Are there any particular features that you find useful for doing your job each day?
   - Are there any aspects of your workflow it improves?

7. What do you find particularly challenging or unintuitive about the Electronic Health Record?
   Prompts:
   - What interface elements that are particularly poorly aligned with your workflow?
   - How effectively does it allow you to capture the data you need to support your work?
   - What are the gaps in data collection?

Experiences with performance feedback

8. What type of feedback do you receive about your performance?

Prompts:

- Does your organization have formal mechanisms to monitor your performance such as dashboards or reports?
- Are there any informal systems you have to get feedback on your performance?
- Do you receive both positive and negative feedback, or is it more common to get one or the other?

9) How does this feedback influence your performance?

10) What do you feel is the most impactful way to provide you feedback about your performance?

Methods of staying up to date with best practice

11) What types of formal activities do you engage in to stay up to date with best practice?

Prompts:

- How do you identify suitable activities to engage in?
- How frequently do you engage in these types of activities?
- To what extent do these activities involve peer or team collaboration?

12) What types of informal activities do you engage in to stay up to date with best practice?

Prompts:

- How do you identify suitable activities to engage in?
- How frequently do you engage in these types of activities?
- To what extent do these activities involve peer or team collaboration?

13) How do you capture these activities as part of your mandatory continuing professional development?

Prompts:

- Are there any activities that are particularly challenging to capture as part of your continuing professional development?
- Are there any continuing professional development requirements that are challenging to find activities to meet?

Electronic Health and Medical Records and Reflective Practice

14) Can you describe any instances where you think the Electronic Health Record could be valuable for supporting your professional learning and reflection?

Prompts:

- How have you used dashboards or data visualizations populated by Electronic Health Record data to understand your performance or your clinical practice?
- How have you used Electronic Health Record reports to reflect on your clinical practice and performance?

15) Are there any types of data collected in the Electronic Health Record that you find particularly useful for understanding your performance?

Prompts:

- How would you use structured information such as prescribing or test results to understand your performance?
- How would you use unstructured information such as details in clinical notes to understand your performance?

16) Are there any scaffolds or features in the Electronic Health Record that prompt you to think about your practice?

Prompts:

- How have you used alerts generated by the Electronic Health Record to understand your performance?
- What sorts of patient presentations are particularly engaging for you to understand your performance?

17) Are there any other scaffolds or feedback mechanisms of engaging with Electronic Health Record data that might prompt you to reflect on your performance?

Prompts

- Would engaging with Electronic Health Record data in the context of an MDT be particularly useful?
- How would you feel about undertaking training that was personalized based on your clinical presentations?

18) What sort of opportunities do you feel an Electronic Health Record could provide to support health professional learning?

19) Do you have any concerns about the use of Electronic Health Records to understand performance or support health professional learning?

20) If you were to design the perfect Electronic Health Record to enable you to understand you clinical practice and learn from that data, what features would it include?

General Comments

21) Do you have any general comments about Performance Feedback in health care?

22) Do you have any general comments about Electronic Health and Medical Records?

23) Do you have any general comments about the role of Electronic Health and Medical Records and health professional learning?
