# Supplementary material for: Technology-Enabled Workplace Learning Through Rethinking Electronic Health Records to Support Performance Feedback: Protocol for a Mixed Methods Study
Source: JMIR Res Protoc. 2025 May 23;14:e66824. doi: 10.2196/66824 (PMC12144470; doi:10.2196/66824)
Supplement: Multimedia Appendix 3 [file resprot_v14i1e66824_app3.pdf]

Technology enabled workplace learning: Rethinking Electronic Health Records to support performance feedback

Workforce Survey Outline

<u>Note to ethics committee:</u> As specific EHR features for prioritization in the Delphi consensus survey are being derived from Phase I interviews, only placeholders are included in this outline to provide an indication of the workforce survey.

STUDY INFORMATION

This survey is presenting features that could be incorporated into Electronic Health and Medical Records to enable these technologies to better support health professional learning. If you choose to complete the survey you will be asked to priorities each feature based on how useful it would be to support your professional learning.

A Participant Information Statement providing detailed information about the study is available here: <INSERT LINK>

[ ] I have read the Participant Information Statement, am aged 18 years or over and consent to being part of this study.

ABOUT YOU

1. What is your health profession?

2. How many years have you been working in this profession?

3. What gender do you identify with?
    - Female
    - Male
    - Trans and/or gender diverse
    - I use a different term (this is inclusive of culturally-relevant terms for the expression and/or identification of your gender, if applicable)
    - Prefer not to say

4. What sector do you currently work in?
    - Public
    - Private
    - Other (please explain your answer)

ELECTRONIC HEALTH RECORD FUNCTIONALITY PRIORITISATION

1. Please indicate your priorities for each of the items below on a 1 – 9 scale, where 1 = lowest importance to you and 9 = highest importance to you.

- Electronic Health Record Feature 1 derived from Phase I interviews
- Electronic Health Record Feature 2 derived from Phase I interviews
- Electronic Health Record Feature 3 derived from Phase I interviews
- Electronic Health Record Feature 4 derived from Phase I interviews
- Electronic Health Record Feature 5 derived from Phase I interviews
- Electronic Health Record Feature 6 derived from Phase I interviews
- Electronic Health Record Feature 7 derived from Phase I interviews
- Electronic Health Record Feature 8 derived from Phase I interviews
- Electronic Health Record Feature 9 derived from Phase I interviews
- Electronic Health Record Feature 10 derived from Phase I interviews

2. Are there any other features you would like Electronic Health Record to have to enable you to utilize them to support your professional learning and reflection about your practice?

………

………

3. Do you have any general comments you would like to make about the use of Electronic Health and Medical records to support health professional learning and practice reflection?

Thank you for completing the survey. If you would like to receive a one page summary of the findings of this research, or are interested participating in a key informant workshop to discuss the redesign of EHRs to better support health professional learning, please follow the link below to provide an email address where you can be contacted by researchers. Your email will kept separate from the responses you have submitted to this survey to ensure your anonymity.

<INSERT LINK>

Please make sure you click submit below as well.
