# Supplementary material for: Technology-Enabled Workplace Learning Through Rethinking Electronic Health Records to Support Performance Feedback: Protocol for a Mixed Methods Study
Source: JMIR Res Protoc. 2025 May 23;14:e66824. doi: 10.2196/66824 (PMC12144470; doi:10.2196/66824)
Supplement: Multimedia Appendix 4 [file resprot_v14i1e66824_app4.pdf]

Technology enabled workplace learning: Rethinking Electronic Health Records to support performance feedback

Key Informant Workshop Guide

<table>
<tr><th>DISCUSSION OUTLINE</th><th>TIME ALLOCATION</th></tr>
<tr><td>Workshop Welcome and Session Setup</td><td>5 minutes</td></tr>
<tr><td>Workforce Survey Results
Overview of results from Electronic Health and Medical Record feature prioritization by health workforce
Questions and feedback from workshop attendees</td><td>15 minutes</td></tr>
<tr><td>Group discussion in Electronic Health and Medical Record functionality redesign
Extent to which current Electronic Health and Medical Records incorporate functionality identified as a priority by the health workforce.
Strategies for increasing visibility and accessibility of current features to improve user experience.
Opportunities for developing new functionality within Electronic Health and Medical Records to support health professional learning and reflective practice.
Feasibility of incorporating ideal feature set into Electronic Health and Medical Records to support</td><td>25 minutes</td></tr>
<tr><td>Linking data-driven learning with Continuing Professional Development
Reflection on how engaging with Electronic Health and Medical Record data to reflect on practice aligns with Continuing Professional Development requirements.
Brainstorming opportunities to harness Electronic health and Medical Record functionality to streamline recording of data-driven health professional learning in Continuing Professional Development systems.</td><td>10 minutes</td></tr>
<tr><td>Workshop Wrap Up:
General Comments from workshop attendees
Next steps for study and workshop close</td><td>5 minutes</td></tr>
</table>
